# Supplementary material for: Causal Relationship between Aging and Anorexia Nervosa: A White-Matter-Microstructure-Mediated Mendelian Randomization Analysis
Source: Biomedicines. 2024 Aug 16;12(8):1874. doi: 10.3390/biomedicines12081874 (PMC11351342; doi:10.3390/biomedicines12081874)
Supplement: Supplementary file 1 [file biomedicines-12-01874-s001.zip › Supplementary Figure/Figure S4/Singe SNP plot of MR analysis for TL to ubm-b-1674.pdf]

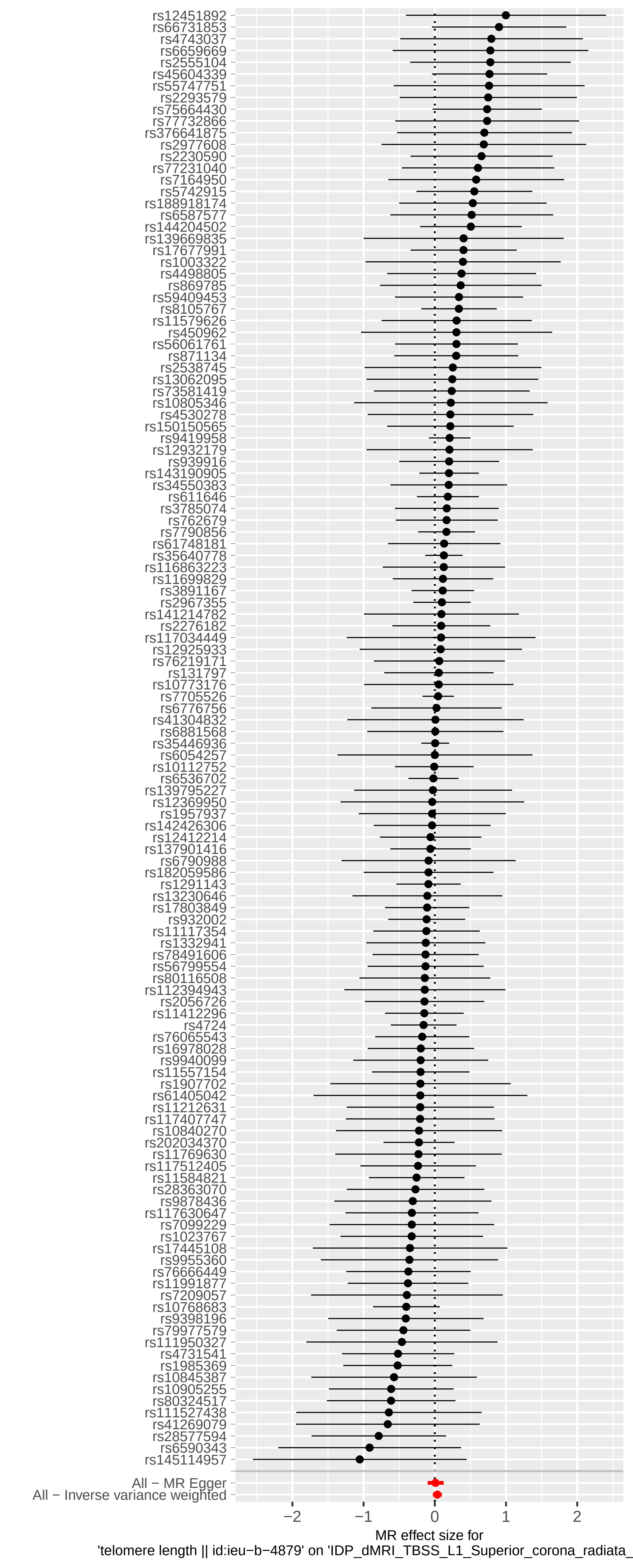
rs12451892
rs66731853
rs4743037
rs6659669
rs2555104
rs45604339
rs55747751
rs2293579
rs75664430
rs77732866
rs376641875
rs2977608
rs2230590
rs77231040
rs7164950
rs5742915
rs188918174
rs6587577
rs144204502
rs139669835
rs17677991
rs1003322
rs4498805
rs869785
rs59409453
rs8105767
rs11579626
rs450962
rs56061761
rs871134
rs2538745
rs13062095
rs73581419
rs10805346
rs4530278
rs150150565
rs9419958
rs12932179
rs939916
rs143190905
rs34550383
rs611646
rs3785074
rs762679
rs7790856
rs61748181
rs35640778
rs116863223
rs11699829
rs3891167
rs2967355
rs141214782
rs2276182
rs117034449
rs12925933
rs76219171
rs131797
rs10773176
rs7705526
rs6776756
rs41304832
rs6881568
rs35446936
rs6054257
rs10112752
rs6536702
rs139795227
rs12369950
rs1957937
rs142426306
rs12412214
rs137901416
rs6790988
rs182059586
rs1291143
rs13230646
rs17803849
rs932002
rs11117354
rs1332941
rs78491606
rs56799554
rs80116508
rs112394943
rs2056726
rs11412296
rs4724
rs76065543
rs16978028
rs9940099
rs11557154
rs1907702
rs61405042
rs11212631
rs117407747
rs10840270
rs202034370
rs11769630
rs117512405
rs11584821
rs28363070
rs9878436
rs117630647
rs7099229
rs1023767
rs17445108
rs9955360
rs76666449
rs11991877
rs7209057
rs10768683
rs9398196
rs79977579
rs111950327
rs4731541
rs1985369
rs10845387
rs10905255
rs80324517
rs111527438
rs41269079
rs28577594
rs6590343
rs145114957
All − MR Egger
All − Inverse variance weighted
−2
−1
0
1
2
MR effect size for
'telomere length || id:ieu−b−4879' on 'IDP_dMRI_TBSS_L1_Superior_corona_radiata_
